# The HSP90 Inhibitor NVP-AUY922 Radiosensitizes by Abrogation of Homologous Recombination Resulting in Mitotic Entry with Unresolved DNA Damage

**DOI:** 10.1371/journal.pone.0035436

**Published:** 2012-04-16

**Authors:** Shane Zaidi, Martin McLaughlin, Shreerang A. Bhide, Suzanne A. Eccles, Paul Workman, Christopher M. Nutting, Robert A. Huddart, Kevin J. Harrington

**Affiliations:** 1 Targeted Therapy Team, Institute of Cancer Research, Chester Beatty Laboratories, London, United Kingdom; 2 Tumour Biology and Metastasis Team, Cancer Research UK Cancer Therapeutics Unit, The Institute of Cancer Research, Haddow Laboratories, Sutton, Surrey, United Kingdom; 3 Signal Transduction and Molecular Pharmacology Team, Cancer Research UK Cancer Therapeutics Unit, The Institute of Cancer Research, Haddow Laboratories, Sutton, Surrey, United Kingdom; 4 The Royal Marsden Hospital, London, United Kingdom; 5 The Royal Marsden Hospital, Sutton, United Kingdom; University of Illinois at Chicago, United States of America

## Abstract

**Background:**

Heat shock protein 90 (HSP90) is a molecular chaperone responsible for the conformational maintenance of a number of client proteins that play key roles in cell cycle arrest, DNA damage repair and apoptosis following radiation. HSP90 inhibitors exhibit antitumor activity by modulating the stabilisation and activation of HSP90 client proteins. We sought to evaluate NVP-AUY922, the most potent HSP90 inhibitor yet reported, in preclinical radiosensitization studies.

**Principal Findings:**

NVP-AUY922 potently radiosensitized cells *in vitro* at low nanomolar concentrations with a concurrent depletion of radioresistance-linked client proteins. Radiosensitization by NVP-AUY922 was verified for the first time *in vivo* in a human head and neck squamous cell carcinoma xenograft model in athymic mice, as measured by delayed tumor growth and increased surrogate end-point survival (p = <0.0001). NVP-AUY922 was shown to ubiquitously inhibit resolution of dsDNA damage repair correlating to delayed Rad51 foci formation in all cell lines tested. Additionally, NVP-AUY922 induced a stalled mitotic phenotype, in a cell line-dependent manner, in HeLa and HN5 cell lines irrespective of radiation exposure. Cell cycle analysis indicated that NVP-AUY922 induced aberrant mitotic entry in all cell lines tested in the presence of radiation-induced DNA damage due to ubiquitous CHK1 depletion, but resultant downstream cell cycle effects were cell line dependent.

**Conclusions:**

These results identify NVP-AUY922 as the most potent HSP90-mediated radiosensitizer yet reported *in vitro*, and for the first time validate it in a clinically relevant *in vivo* model. Mechanistic analysis at clinically achievable concentrations demonstrated that radiosensitization is mediated by the combinatorial inhibition of cell growth and survival pathways, ubiquitous delay in Rad51-mediated homologous recombination and CHK1-mediated G_2_/M arrest, but that the contribution of cell cycle perturbation to radiosensitization may be cell line specific.

## Introduction

HSP90 is a ubiquitously expressed molecular chaperone which exists as part of a larger complex consisting of HSP70 and co-chaperones such as Cdc37, p23, AHA1, Hip and Hop [1], [2]. The normal function of HSP90 in cellular homeostasis is the conformational maintenance of a pool of client proteins and, as such, it is essential for their sustained activity. The finding that HSP90 maintains the stability and activity of a range of oncoproteins which give rise to the malignant phenotype [3] has resulted in the concept of chaperone addiction; i.e. tumor cells in which continued oncogenic activity is reliant on the underlying molecular chaperone machinery of the cell [4]. Key amongst HSP90's oncogenic clientele are receptor tyrosine kinases, cyclin-dependent kinases, hypoxia-linked factors and telomerase [3], [4]. Many of these client proteins have been identified to play key roles in cell cycle arrest, DNA damage repair and apoptosis in response to radiotherapy [5], [6]. This has made HSP90 an intriguing target in the field of radiosensitization [7].

The great advantage of HSP90 targeted therapies is the simultaneous, combinatorial depletion of many potentially oncogenic factors by a single therapeutic agent. Early HSP90 inhibitors were based on the natural compound geldanamycin, which gave rise to a number of analogs with improved pharmacological properties, including the first-in-class analog 17-AAG. Preclinical HSP90 mediated radiosensitization has been reported with both geldanamycin and its derivatives (17-AAG and 17-DMAG) [8], [9] as well as the orally bioavailable PU3 purine scaffold derivative BIIB021 [10]. Geldanamycin family compounds have been shown to radiosensitize a diverse array of tumor-derived cell lines *in vitro*, including squamous cell [11], prostate [8], [9], [12], lung [13], colorectal [13]–[15], cervical [16], [17], bladder [14] and pancreatic carcinomas [9], glioma [8], [17] and melanoma [14]. In addition, radiosensitization of human vascular endothelial cells has been reported [18].

*In vivo* radiosensitization has been shown in human cervical [16], prostate [12] and head and neck squamous cell carcinoma (HNSCC) [10] tumor xenograft models. Response has been shown to be dependent on cell division, since fibroblasts that originally were not radiosensitized by geldanamycin or 17-AAG became sensitive upon transformation by HPV16 E7 or E6 [14], [16]. The geldanamycin derivatives 17-AAG and 17-DMAG have, thus far, proven useful in providing mechanistic insights, preclinical and clinical validation of biomarkers of HSP90 inhibition and identification of other beneficial effects such as anti-angiogenic properties [19], [20].

Until now, the success of 17-AAG (tanespimycin) in phase II clinical trials has been limited. While phase I trials showed signs of clinical activity [21]–[23], phase II trials have been less conclusive, with evidence of response observed in metastatic melanoma [24] but not for metastatic prostate [25] or papillary and clear cell renal carcinomas [26]. Phase I studies of 17-DMAG have shown HSP72 induction and promising signs of clinical activity [27]. In this regard, the need for HSP90 inhibitors of greater potency and efficacy is evident and has given rise to a number of synthetic alternatives, one of the most promising of which is NVP-AUY922 (VER-52296). This agent is a fully synthetic isoxazole resorcinol-based HSP90 inhibitor and is the most potent NH_2_-terminal HSP90 inhibitor yet described [20], [28]. NVP-AUY922 has been shown to have anti-proliferative effects *in vitro* against a panel of breast cancer cell lines and primary cultures [29], multiple myeloma [30], prostate [20], [28], colon, melanoma, glioma [28], [31] and HUVEC cell lines [20]. Efficacy as a single agent has been seen *in vivo* in BT-474 breast [29], HCT116 colorectal [28] and U87MG glioblastoma [31] xenografts in mice.

NVP-AUY922 has been shown to overcome a number of limitations associated with 17-AAG, exhibiting selectivity for HSP90, increased solubility, an absence of the hepatotoxicity-linked quinone moiety and independence of 17-AAG-linked NQO1 metabolism [20]. Also important is the substantially increased potency, with a 60-fold decrease in IC_50_ values for fluorescence polarisation binding assays and 10-fold decrease in HCT116 GI_50_ concentrations compared with 17-AAG [28].

In this report, we describe the ability of NVP-AUY922 to radiosensitize cervical, colorectal and HNSCC cell lines with greater potency than any previously reported HSP90 inhibitor. We also report confirmation for the first time of radiosensitization by NVP-AUY922 *in vivo*. Mechanistic analysis *in vitro* indicates that radiosensitization is likely to be combinatorial in nature, with inhibition of growth signalling, radiation-induced DNA damage repair by homologous recombination and perturbation of cell cycle progression into mitosis all likely contributing factors.

## Materials and Methods

### Cell Culture Conditions

HCT116 (colorectal carcinoma) and HeLa (uterine cervical carcinoma) were obtained from the ATCC. LICR-LON-HN3 and LICR-LON-HN5 (HNSCC) cell lines, from here on referred to as HN3 and HN5 for brevity, were obtained from stocks in the Targeted Therapy Lab, The Institute of Cancer Research, London, United Kingdom. Cells were cultured in DMEM (Invitrogen, Paisley, UK) supplemented with 2 mM L-glutamine and 1% penicillin/streptomycin in a humidified incubator at 37°C with 5% CO_2_. Cells were subcultured at 80–90% confluency and routinely tested for the presence of mycoplasma using the eMyco PCR kit from IntroBio (Seongnam-Si, South Korea). Cell line stocks were authenticated by short tandem repeat profiling carried out by Bio-Synthesis Inc. (Texas, US).

### Compounds and Irradiation

NVP-AUY922 was kindly donated by Novartis in the form of the mesylated salt and was reconstituted in DMSO before storage at −20°C. 5% dextrose was used as a vehicle for *in vivo* intraperitoneal delivery. Irradiation was carried out using an AGO 250 kV X-ray machine (AGO, Reading, UK).

### Clonogenic Assay

Cell survival was measured by colony formation assay. Cells were trypsinised, diluted and counted before seeding in 6-well dishes at varying cell densities. After cells had attached they were treated with NVP-AUY922 or DMSO control for 24 h before irradiation with 0–8 Gy. After 10–14 days, colonies were fixed and stained in 7% ethanol, 0.5% crystal violet, with colonies containing more than 50 cells counted. Colony counting was performed manually and using the Oxford Optronics Colcount system (Oxford, UK). Percent cell survival was estimated by normalizing data to drug-free unirradiated control. Surviving fraction was derived using the formula: Number of Colonies/Number of cells plated x PE (where PE equals Number of Colonies/Number of cells plated in drug-free unirradiated control).

### *In Vivo* Human Xenograft Model and Ethics Statement

All animal studies were conducted in accordance with NCRI (National Cancer Research Institute) guidelines [32]. All animal research was reviewed and approval under the ethical review process by the Institute of Cancer Research Ethics Committee. These experiments were performed under the authority given by UK Home Office Project License PPL 70/6890. HN3 cells were implanted by injection of 3×10^6^ cells into the right flank of 6-week old female CD-1 nude mice (Charles River, UK). Mice were randomised into experimental groups after tumors had achieved a maximum diameter of 5–8 mm with differing tumor volumes evenly distributed between groups (mean starting volumes: control 116.1±44.2; NVP-AUY922 10 mg/kg only 130.1±66.9; fractionated radiation only 133.7±41.6; NVP-AUY922 10 mg/kg plus radiation 139.7±36.6). NVP-AUY922 in 5% dextrose was injected intraperitoneally in a volume of 200 µl at the desired time points and irradiation carried out post-NVP-AUY922 administration. Briefly, animals were anaesthetized by intraperitoneal injection of 100 µl of a 1∶1∶4 mixture of Hypnorm (fentanyl citrate 0.315 mg/mL, fluanisone 10 mg/mL) from Janssen-Cilag Ltd (High Wycombe, UK), Hypnovel® (midazolam 5 mg/mL) from Roche (Welwyn Garden City, UK) and water for injection BP from Fresenius Health Care Group (Basingstoke, UK). Mice were positioned under lead shielding with subcutaneous tumors only exposed to localized radiation. Perpendicular diameter measurements of each tumor were taken twice weekly with volumes calculated using the formula (width x width x length)/2. Tumors were followed until the maximum diameter reached 15 mm (humane end point), or ulceration. Tumor and liver samples, with liver as a representative normal tissue sample, were snap frozen following harvesting for subsequent western blot analysis.

### Western Blotting and Antibodies

Cells were seeded in 10 cm dishes and allowed to recover for 24 h before NVP-AUY922 addition. Media, washes and cells were subsequently harvested with PBS containing 2 mM Na_3_VO_4_ and centrifuged at 1,200 rpm before lysis in RIPA buffer containing 50 mM Tris.HCl pH 7.5, 150 mM NaCl, 1% NP-40, 0.5% deoxycholate and 0.1% SDS. Samples were thawed on ice, centrifuged at 14,000 rpm for 20 min at 4°C and supernatants quantified by BCA assay from Pierce (Leicestershire, UK). 20–30 µg total protein were separated by reducing 10% SDS-PAGE, transferred to PVDF membrane (GE Healthcare, Bucks, UK), blocked with 5% non-fat dry milk in PBS for 1 h and probed with the following primary antibodies in 5% BSA and 0.1% Tween-20: rabbit anti-HSP72 from Stressgen (Exeter, UK), rabbit anti-ErbB2, rabbit anti-cRAF from Santacruz (CA, USA), rabbit anti-pErbB2, rabbit anti-pCHK1, rabbit anti-CHK1, rabbit anti-pAKT, rabbit anti-AKT and rabbit anti-pH2ax were all purchased from Cell Signalling (MA, USA), rabbit anti-p-histone H3 was purchased from Upstate-Millipore (UK). Loading was quantified using mouse anti-γ-tubulin from Sigma (Poole, UK). Secondary antibodies used were goat anti-mouse IgG HRP from Jackson Immunoresearch (Suffolk, UK) and donkey anti-rabbit IgG from GE Healthcare. Chemiluminescent detection was carried out using immobilon western substrate from Millipore according to the manufacturer's instructions. *In vivo* samples were processed by homogenisation in RIPA buffer using a Precellys®24 homogeniser from Bertin Technologies (Montigny, France) before SDS-PAGE and western analysis as for *in vitro* samples.

### DNA Damage Repair

Cells were seeded at 1×10^5^ on 35 mm dishes with an inner 10 mm glass well from Mattek (MA, USA) and allowed to recover for 24 h. Cells were treated with NVP-AUY922 or DMSO only for 24 hours and irradiated with 4 Gy. At 4 and 24 hours samples were fixed in 10% formalin for 30 minutes at room temperature. Cells were subsequently washed, permeabilised in 0.2% Triton X-100, treated with DNase I from Roche (West Sussex, UK) at 37°C for 1 hour, rinsed in PBS and blocked in 1% BSA, 2% FCS in PBS for 1 hour. Cells were stained with rabbit anti-phopho-H2ax (Cell Signalling) and mouse anti-RAD51 (Genetex, CA, USA) and visualized with Alexafluor 488 goat anti-rabbit secondary and Alexafluor 546 goat anti-mouse (Invitrogen, Paisley, UK). Nuclei were counterstained with TO-PRO-3 iodide from Invitrogen and nuclear dsDNA damage visualised using a Zeiss LSM710 inverted confocal microscope (Carl Zeiss, Jena, Germany).

### Nuclear Volume and Micronuclei

Cells were seeded at 1×10^5^ in 6 cm or Mattek dishes and allowed to recover for 24 h. NVP-AUY922 or DMSO vehicle was added for 24 hours before irradiation. Cells were fixed in 10% formalin for 30 min, rinsed in PBS and nuclei stained for 5 min with 4′, 6-diamidino-2-phenylindole (DAPI) from Sigma (Poole, UK). Z-stack confocal microscopy acquisition was used in conjunction with Volocity v5.2 (Perkin Elmer) image processing and analysis software to quantify nuclear volume as well as the presence of multinucleated cells and micronuclei.

### Mitotic Index and Cell Cycle Analysis

Cells were seeded in 10 cm dishes and allowed to recover for 24 h. Vehicle or NVP-AUY922 was added at the concentrations indicated. After 16 h cells were irradiated with 4 Gy. At time points post-irradiation, media and trypsinised cells were harvested and immediately processed on ice, washed in PBS, resuspended in 1 ml of ice-cold PBS at 5×10^5^ cells/ml and syringed into 4 ml of ice-cold ethanol while vortexing. After fixation, cells were washed in PBS, blocked in 1% BSA, 2% FCS in PBS for 1 hour before staining with 1 µg/ml rabbit anti-phospho- histone H3 from Millipore-Upstate (Watford, UK) overnight at 4°C followed by goat anti-rabbit Alexafluor-488 secondary for 2 hours at room temperature. Cells were resuspended in 500 µl of 100 µg/ml RNase A and 10 µg/ml propidium iodide (Sigma), before analysis by flow cytometry using an LSR II from BD Biosciences (Oxford, UK).

### Statistical Analysis

Graphpad prism (version 5.0c) was used to carry out statistical analysis. Paired or unpaired two-tailed student t-test was selected for parametric analysis. Analysis of surrogate end point data was carried out using the Mantel-Cox log-rank test.

## Results

### Radiosensitivity induced by NVP-AUY922 *in vitro*

**Figure 1:**
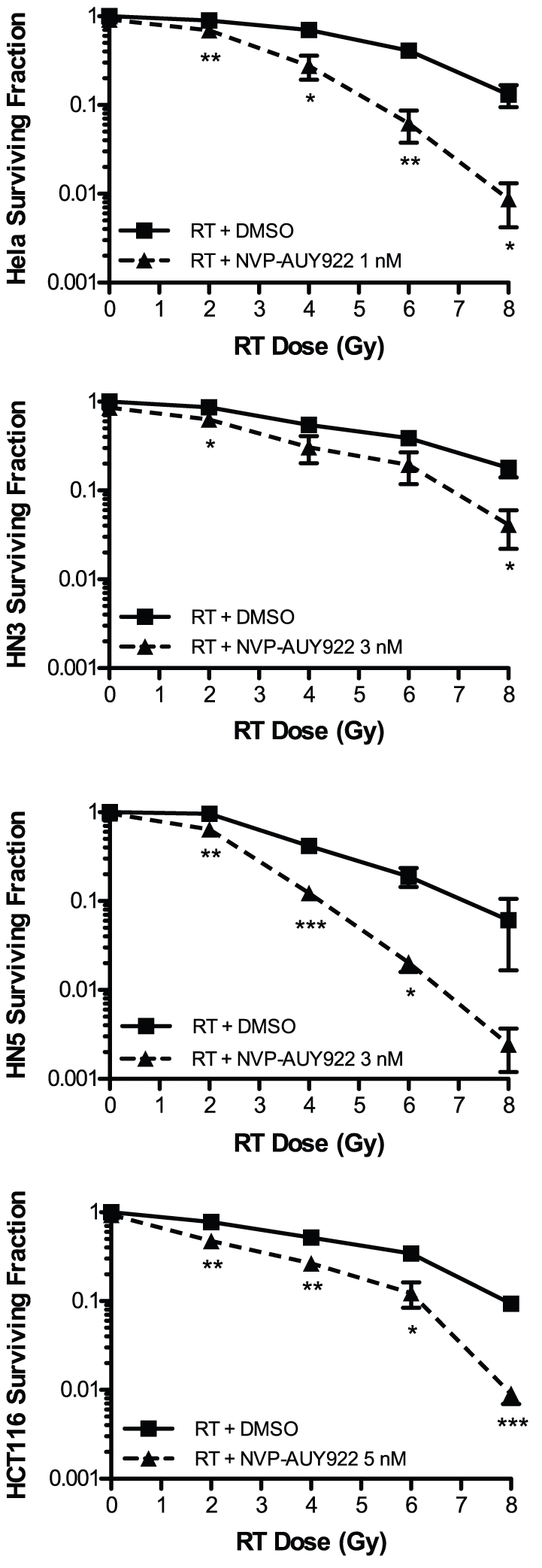
NVP-AUY922 decreases clonogenic survival due to ionising radiation *in vitro*. Clonogenic assay was utilised to assess radiosensitization by NVP-AUY922 *in vitro* against the uterine cervical carcinoma and colorectal carcinoma cell lines HeLa and HCT116 and the HNSCC cell lines HN3 and HN5. After plating at an appropriate seeding density and allowed to attach, cells were exposed to NVP-AUY922 at the concentrations indicated per cell line for 24 h to facilitate downstream effects of HSP90 inhibition to occur. Cells were subsequently exposed to ionising radiation at the doses indicated. Colony formation was determined at 10 to 14 days after irradiation and surviving fractions calculated relative to plating efficiencies for vehicle only non-irradiated cells. Data ± SEM with statistical analysis carried out by two-tailed t-test between surviving fractions at each radiation dose, *p<0.05, **p<0.01, ***p<0.001.

The ability of NVP-AUY922 to radiosensitize HeLa (uterine cervical carcinoma), HN3, HN5 (HNSCC) and HCT116 (colorectal carcinoma) cells *in vitro* was assessed by clonogenic assay (Fig. 1). These cell lines were selected as representative of tumor types for which radiotherapy is a curative treatment option in the clinic as well as the prevalence of common oncogenic molecular characteristics of relevance to HNSCC and radioresistance (EGF-R overexpression, HN5 and HN3; HPV status, HeLa; activated kRAS and PI3K catalytic subunit, HCT116). The highest concentration of NVP-AUY922 alone which did not reduce clonogenic survival was determined for each cell line. For all four cell lines, these concentrations were in the low nanomolar range: 1 nM for HeLa, 3 nM for HN3 and HN5, and 5 nM for HCT116. Radiation survival curves generated with and without NVP-AUY922 (Fig. 1) show statistically significant radiosensitization for all four cell lines in the presence of NVP-AUY922 (p-values at 2 Gy between drug free and treated are, HeLa p = 0.004, HN3 p = 0.0157, HN5 p = 0.0023 and HCT116 p = 0.0094). Radiosensitization by 17-AAG has been reported in the literature within the range of 30–200 nM (see discussion). Consistent with this, radiosensitization by 17-AAG in the panel of four cell lines used was observed at 100 nM (Fig. S1A). This indicates that NVP-AUY922 is a substantially more potent radiosensitizer than 17-AAG *in vitro* (Fig. S1A), in the low nanomolar range and at clinically relevant radiation doses, i.e. 2 Gy.

### NVP-AUY922 radiosensitizes *in vivo* in a HNSCC human xenograft model

**Figure 2:**
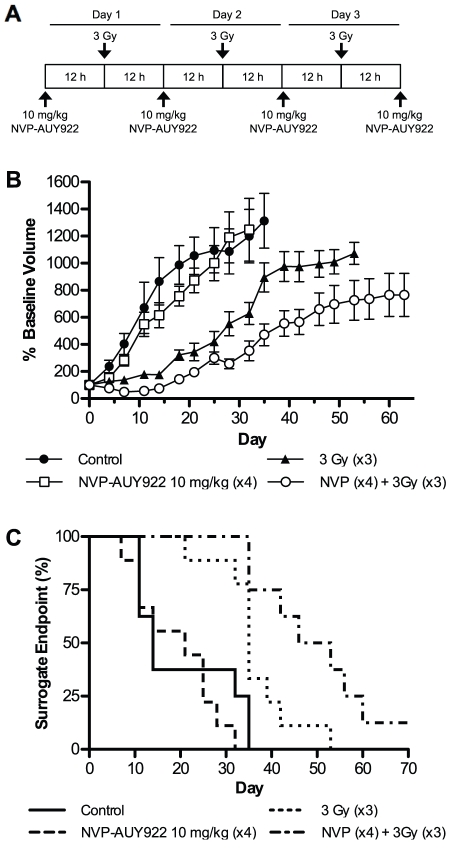
Sub-optimal dosing of NVP-AUY922 delays tumor growth and increases time to surrogate endpoint *in vivo* in conjunction with ionising radiation. Radiosensitization due to NVP-AUY922 was assessed *in vivo* in a human xenograft model. HN3 cells were allowed to achieve a tumor volume of 5–8 mm after implantation in the right flank and evenly distributed into four treatment groups with matching average tumor volumes; vehicle only control (n = 8); NVP-AUY922, four doses of 10 mg/kg each (n = 9); 9 Gy ionising radiation fractionated in three doses of 3 Gy (n = 9); NVP-AUY922 and ionising radiation (n = 8). (A) Scheduling of four rounds of 10 mg/kg NVP-AUY922 or vehicle only was administered by interperitonal injection, followed by fractioned tumor-targeted radiotherapy of 3 Gy each to a cumulative total of 9 Gy on days one to three. Tumor sizes were measured every 3–4 days with resulting percentage baseline tumor volume (B) and percentage surrogate endpoint (C) shown. Data represents ± SEM. Mantel-Cox log-rank test carried out for statistical analysis of surrogate endpoint data (p = 0.0002).

To assess the efficacy of NVP-AUY922 as a radiosensitizer in HNSCC *in vivo*, a HN3 human xenograft model in CD-1 nude mice was selected. Due to the routine clinical administration of radiation in fractions, 10 mg/kg NVP-AUY922 was given intraperitoneally on four consecutive days, followed by three fractionated radiation doses of 3 Gy 12 h after drug administration on days one to three (Fig. 2A). Ten mg/kg NVP-AUY922 was selected as a suboptimal dose based on previously reported activity *in vivo* [29]. Tumors were measured every 2–3 days with the resulting measurements expressed relative to baseline tumor volumes at the commencement of treatment (Fig. 2B). A maximum tumour diameter of 15 mm was used as a humane endpoint and surrogate indicator of survival (Fig. 2C). Tumor volumes unadjusted for baseline are shown in supporting information (Fig. S1B).

NVP-AUY922 10 mg/kg alone had negligible effect in reducing tumor volume over the duration of the experiment, with growth rates similar to those observed in the vehicle treated control group (Fig. 2B). As expected, a significant delay in tumor growth was observed in the fractionated radiation only group, up until approximately day 15–20. At this point, tumor growth accelerated with the percentage reaching surrogate survival endpoint rapidly increasing from day 35 onwards. The radiation plus NVP-AUY922 group was the only group in which a drop in tumor volume was observed at any stage, this was maintained until day 18 at which point tumor volume had recovered to baseline levels. From this point, growth for radiation plus NVP-AUY922 was significantly slower than that observed for radiation alone (Fig. 2B). This was mirrored in the significantly increased time to surrogate survival endpoint (Fig. 2C) of the NVP-AUY922 plus radiation group compared to fractionated radiation alone (p = 0.0002). The NVP-AUY922 only group exhibited an increase in median time to surrogate survival endpoint from 14 to 21 days, while in conjunction with fractionated radiation median time to surrogate survival endpoint increased by 14.5 days over fractionated radiation alone (35 to 49.5 days) with one animal cured at the termination of the experiment. The plateau effect observed after 35 days was due to loss of mice with rapidly growing tumours having reached the surrogate survival humane end point (Fig. 2C), as stipulated in UK NCRI animal welfare guidelines [32].

### Biomarker response to NVP-AUY922

**Figure 3:**
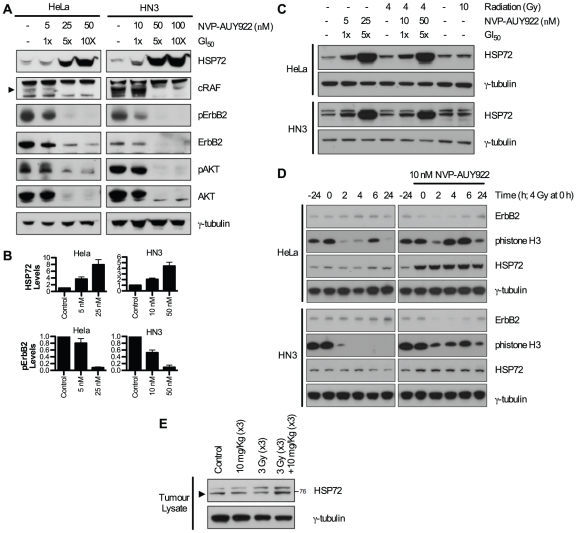
Molecular signature of NVP-AUY922 in combination with radiation. (A) To confirm depletion of HSP90 client proteins linked to radioresistance, levels were assessed *in vitro* in HeLa and HN3 cell lines by western blot. Cells were treated with vehicle only, or the concentrations of NVP-AUY922 indicated for 24 h, followed by reducing SDS-PAGE of 30 µg whole cell lysate and western blot detection of the HSP90 client proteins shown. (B) Levels of HSP72 28 h post NVP-AUY922 treatment and phospho-ErbB2 24 h post NVP-AUY922 treatment were determined by densitometry of three independent experiments (see Fig. S2A). (C) HeLa and HN3 cells were pre-treated with NVP-AUY922 for 24 h at the concentrations indicated. Cells were irradiated with 4 Gy or 10 Gy and 4 h later whole cell lysates harvested and probed for HSP72 by western blot. (D) Cells were pre-treated with 10 nM NVP-AUY922 for 24 h with 4 Gy of radiation delivered at the 0 hour time point. At the subsequent time points indicated, whole cell lysates were harvested and probed for total-ErbB2, phospho-histoneH3 and HSP72. (E) Tumor samples were harvested at the point of final irradiation to determine response to NVP-AUY922 *in vivo*. Tissue samples were homogenised and 30 µg of protein probed for HSP72 by reducing SDS-PAGE and western blot. γ-tubulin was probed as loading control in all blots shown.

It has previously been reported that successful HSP90 inhibition by both 17-AAG [33] and NVP-AUY922 [20] generates a molecular signature characterised by induction of HSP72 and depletion of HSP90 client proteins. Depletion of radioresistance-linked client proteins has been shown to occur at concentrations correlating to radiosensitization. Therefore, NVP-AUY922-mediated depletion of AKT, ErbB2 and cRAF was investigated in HeLa and HN3 cells (Fig. 3A).

Biomarker response in both HeLa and HN3 cells *in vitro* was observed to be similar at comparable GI_50_ doses. HSP72 was clearly upregulated at 1×GI_50_ in both HeLa and HN3 cancer cell lines and substantially upregulated at 5–10×GI_50_ doses. Concurrently, cRAF (band indicated by arrow), a component of the RAS-RAF-MEK-ERK pathway and total- and phospho-AKT were minimally decreased at 1×GI_50_ with substantial depletion observed at 5–10×GI_50_ values. Total- and phospho-ErbB2 displayed the greatest sensitivity to HSP90 inhibition by NVP-AUY922, with a clear depletion observable at 1×GI_50_ concentrations in both HeLa and HN3 cell lines. Tumor samples were harvested at the point of administration of the final radiation fraction to determine biomarker response *in vivo*. Increased HSP72 was clearly visible in tumors of the NVP-AUY922 and radiation combined group (Fig. 3B). Investigations into *in vitro* HSP72 upregulation by radiation combined with NVP-AUY922 indicated minor upregulation of HSP72 at 4 h post-radiation alone, in HeLa, HN3 (Fig. 3C) and HN5 (Fig. S2) cell lines. Minor fluctuations in HSP72 due to radiation only were observed in time course analysis in HeLa and HN3 cancer cells up to 24 and 28 h post-radiation (Fig. 3D; Fig. S2C). However, any increases observed were insignificant when compared to the increase in HSP72 observed in the presence of HSP90 inhibitor (Fig. 3D), with NVP-AUY922 shown clearly to elicit ErbB2 depletion and drive aberrant mitotic progression.

### NVP-AUY922 delays Rad51 foci formation and subsequent resolution of radiation-induced DSB repair

**Figure 4:**
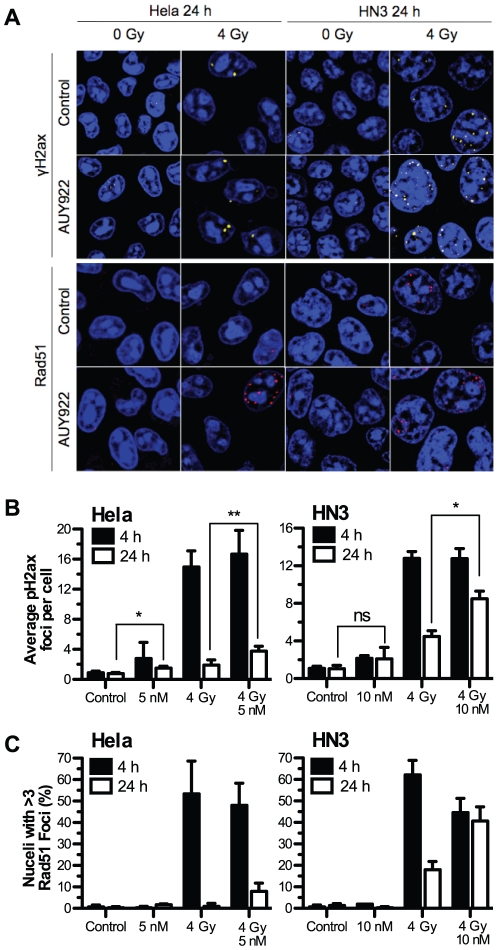
NVP-AUY922 delays radiation induced Rad51 foci formation and DNA damage repair. HeLa and HN3 cells were plated in glass bottom dishes and after attachment exposed to NVP-AUY922 or DMSO control. 24 h post drug-treatment cells were mock irradiated or irradiated with 4 Gy, 4 h and 24 h post-irradiation cells were fixed and stained for dsDNA breaks using anti-phospho-H2ax and Rad51 focal formation with TO-PRO-3 as nuclear counter stain. (A) Representative images of remaining foci at 24 h are shown for HeLa and HN3 cells, area shown represents 60 µm by 60 µm. (B) Mean phospho-H2ax foci per-cell for both HeLa and HN3 at 4 h and 24 h post irradiation was quantified in three independent experiments consisting of phospho-H2ax counts for 150 cells per experiment. (C) Rad51 foci were quantified in two independent experiments with nuclei containing greater than 3 Rad51 foci scored as positive. Values stated are ± SEM for phospho-H2ax, ± SD for Rad51. Statistical analysis by student t-test between groups indicated, *p<0.05, **p<0.01.

HSP90 inhibition by the geldanamycin class of inhibitors has been shown previously to interfere with double-strand DNA break (DSB) resolution [15], [17]. Due to the more selective nature of NVP-AUY922 as an HSP90 inhibitor, we sought to verify the ability of NVP-AUY922 to abrogate effective DSB repair and impair Rad51-mediated homologous recombination. Quantification of phospho-H2ax (pH2ax) foci, a marker of DNA DSBs, and focal formation of the homologous recombination protein Rad51 were carried out by confocal microscopy. Representative images of pH2ax and Rad51 focal staining at 24 h are shown for both HeLa and HN3 cell lines (Fig. 4A) alongside quantification of pH2ax (Fig. 4B; Fig. S3A) and Rad51 foci (Fig. 4C; Fig. S3B) in HeLa, HN3, HN5 and HCT116 cell lines.

NVP-AUY922 delayed resolution of radiation-induced DSB repair in all four cell lines tested, as shown by increased residual pH2ax foci at 24 h. NVP-AUY922 alone, at GI_50_ concentrations in all four cell lines (Fig. 4B; Fig S3), resulted in a small increase in pH2ax focal formation at 4 h. Foci remained constant and did not increase from 4 h to 24 h. In HeLa (Fig. 4B), HN5 and HCT116 (Fig. S3), the addition of NVP-AUY922 to 4 Gy irradiation indicated a small but statistically insignificant increase in focal formation at 4 h. Tracking the decrease in pH2ax foci between 4 and 24 h as indicative of DSB resolution, NVP-AUY922 reduced the resolution of radiation-induced DSB in all four cell lines tested. pH2ax foci remaining at 24 h due to the combination of NVP-AUY922 and radiation increased over radiation alone from 1.9 to 3.8 foci per cell in HeLa, 4.5 to 8.5 foci per cell in HN3 (Fig. 4B), 5.1 to 7.4 foci per cell in HN5 and 1.0 to 5.6 foci per cell in HCT116 (Fig. S3). Co-inciding with this abrogation of radiation-induced DSB repair, NVP-AUY922 was shown to slow the progression of Rad51-focal formation in all four cell lines. NVP-AUY922 caused a decrease in Rad51 positive nuclei 4 h post radiation, scored as greater than 3 Rad51 foci, in all four cell lines tested (Fig. 4C; Fig. S3B). At 24 h post irradiation, the opposite was true, with an increase in Rad51 positive cells observed due to the addition of NVP-AUY922. This increase at 24 h was from 1 to 8% in HeLa cells, 18 to 40.7% in HN3 cells, 9.6 to 24.3% (Fig. 4C) in HN5 cells and zero to 9.2% in HCT116 cells (Fig. S3B).

### Increased micronuclei and nuclear volume due to irradiation in the presence of NVP-AUY922

The definition of mitotic catastrophe [34], [35] stipulates that attempts at aberrant chromosome segregation are followed by either caspase-2 dependent apoptosis or cells failing in apoptotic execution likely to result in aneuploidy. In order to assess these facets of mitotic catastrophe in respect of NVP-AUY922, micronuclei and nuclear volume at 48 h were quantified as morphological measures of aberrant chromosomal segregation and aneuploidy, respectively.

**Figure 5:**
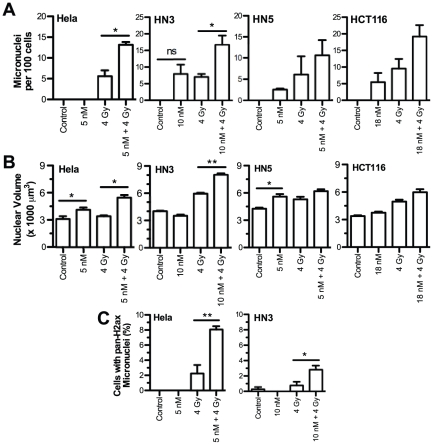
NVP-AUY922 in combination with radiation increases micronuclei formation and nuclear volume. The cell lines indicated were treated with vehicle only or NVP-AUY922 for 24 h before mock irradiation or irradiation with 4 Gy. 48 h post-irradiation cells were fixed and nuclei stained with DAPI. (A) Nuclear morphology was imaged by Z-stack confocal microscopy and the presence micronuclei of quantified for 100 cells. (B) 3-dimensional nuclear topography was constructed using Volocity image processing and analysis software and utilised to quantify increased nuclear volume due to ionising radiation and NVP-AUY922 in 150 nuclei. (C) Confocal images at 24 h used for phospho-H2ax foci formation in figure 4 were quantified for pan-phospho-H2ax staining present in micronuclei. Values shown for quantification of a minimum of 100 nuclei. All values shown are for three independent experiments ± SEM with statistical analysis by student t-test between the groups indicated, *p<0.05, **p<0.01.

Quantification of micronuclei expressed as number of micronuclei per 100 cells (Fig. 5A) revealed that NVP-AUY922 in combination with radiation resulted in increased micronuclei formation over radiation alone which was greater than additive in all four cell lines, i.e. the increase observed when both were combined was greater than that observed were the increase due to each in isolation to be added together. Drug only increases in micronuclei over controls were zero for HeLa, 8 for HN3, 2.6 for HN5 and 5.5 for HCT116. The addition of GI_50_ concentrations of NVP-AUY922 to radiation over radiation alone resulted in an increase in micronuclei formation from 5.6 to 13.2 in HeLa cells, 7 to 16.7 in HN3 cells, 6.1 to 10.6 in HN5 cells and 9.5 to 19.1 in HCT116 cells. Analysis of micronuclei containing pan-pH2ax staining at 24 h post irradiation showed greater than additive increases due to NVP-AUY922 combined with radiation in all four cell lines (Fig. 5C; Fig. S4). NVP-AUY922 alone did not alter the level of cells positive for pH2ax-containing micronuclei from those observed in controls.

Quantification of nuclear volume 48 h post irradiation (Fig. 5B) revealed nuclear volume increased significantly for HeLa and HN5 cells due to NVP-AUY922 alone while increases were minimal in HN3 and HCT116 cells. In HN5 cells, radiation and NVP-AUY922 combined resulted in an increase above radiation alone in HN5 cells, while HeLa, HN3 and HCT116 cell lines all showed a greater than additive increase in nuclear volume due to the combination of NVP-AUY922 and radiation.

### NVP-AUY922 inhibits CHK1-mediated G_2_ arrest in the presence of radiation-induced DNA DSBs

**Figure 6:**
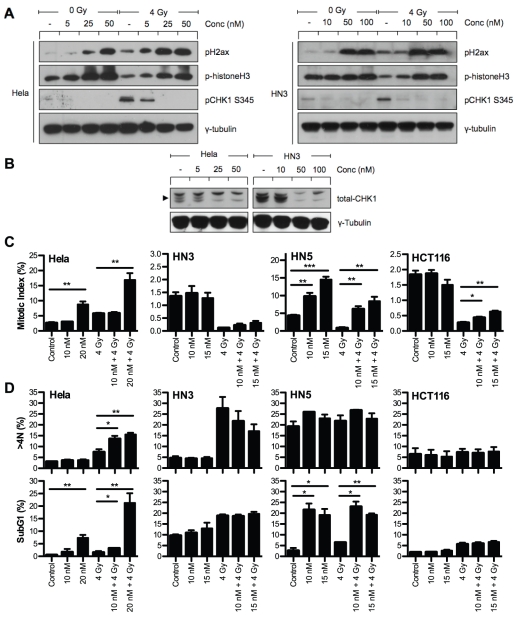
NVP-AUY922 abrogates CHK1-mediated G_2_/M arrest in the presence of DNA damage inducing cell cycle disruption in p53 null cell lines only. The ability of NVP-AUY922 to abrogate G_2_ arrest in the presence of dsDNA damage was investigated by western blot and FACS analysis in all four cell lines (p53 status: HCT116 p53wt, Hela HPV-positive, HN5 and HN3 p53 mutant). (A) HeLa and HN3 cells were pre-treated with NVP-AUY922 for 16 h at the concentrations indicated. Cells were then mock irradiated or irradiated with 4 Gy and 4 h later whole cell lysates harvested, 30 µg protein resolved on 10% SDS-PAGE then probed for phospho-H2ax, phospho-histone H3 and phospho-CHK1 by western blot. (B) The effect of NVP-AUY922 on depletion of total-CHK1 was also investigated in unirradiated cells with γ-tubulin probed as loading control. (C+D) Cells were exposed to the NVP-AUY922 concentrations indicated for 16 h before mock irradiation or irradiation with 4 Gy. Cells were fixed 9 h and 48 h post-irradiation before staining for the mitotic marker p-histone H3 and DNA content with propidium iodide. SubG_1_ and >4N populations were quantified by FACS analysis. Data represents ± SEM of three independent experiments each recording at least 10,000 events. Statistical analysis carried out by two-tailed t-test between the groups indicated, *p<0.05 **p<0.01 ***p<0.001.

HSP90 inhibitors have been shown to inhibit radiation-induced G_2_ arrest [12], [15]. Therefore, we sought to verify the ability of NVP-AUY922 to drive aberrant mitotic entry in the presence of radiation-induced DNA damage (Fig. 6A) and downstream consequences with respect to p53 status (HCT116 p53wt, Hela HPV-positive, HN5 and HN3 p53 mutant). In both HeLa and HN3 cells, radiation only at 4 h resulted in increased pH2ax (DNA damage), increased pS345 CHK1 (G2/M checkpoint arrest) and decreased phistone H3 (mitotic marker). In both of these cell lines, NVP-AUY922 abrogated radiation-induced pS345 CHK1 with a concomitant increase in phistone H3, indicating aberrant mitotic entry in the presence of radiation-induced DNA damage. Decreased pS345 CHK1 levels at higher drug concentrations corresponded, in part, to loss of total-CHK1 (Fig. 6B).

Quantification of phistone H3 positive cells (mitotic index) by flow cytometry showed NVP-AUY922 abrogated the radiation-induced decrease in mitotic index in all four cell lines (Fig. 6C). Two distinct phenotypes were identifiable, HeLa and HN5 cell lines showed a significant increase in mitotic index due to NVP-AUY922 alone which was maintained even after radiation, while no increase due to drug alone was observed for HN3 and HCT116 and abrogation of G2/M arrest was less significant than for HeLa and HN5.

Downstream analysis of cell cycle perturbation due to CHK1 abrogation revealed markedly disparate outcomes. HeLa (HPV-positive) cells 48 h post-irradiation showed an increase in >4N cells from 7.4% in 4 Gy only samples to 14.5 and 15.5% due to the addition of 10 and 20 nM NVP-AUY922, respectively (Fig. 6D), with drug alone causing no increase. 20 nM NVP-AUY922 in combination with radiation resulted in an apoptotic subG_1_ population substantially greater than that observed for 4 Gy or drug only. Increasing subG_1_ and >4N was at the expense of G_1_ but not G2 or S phase (Fig. S5), indicating failed mitotic progression. HCT116 (p53 wild-type), the most resistant cell line to NVP-AUY922 in our panel, showed little cell cycle perturbation by NVP-AUY922 at 48 h in the presence or absence of radiation except for accumulation in G_1_ at the expense of S phase, consistent with G_1_ arrest (Fig. 6D; Fig. S5B).

The cell cycle profiles of HN3 and HN5 differ from HeLa or HCT116 in that they are both highly affected by either radiation alone (HN3) or NVP-AUY922 alone (HN5). HN3 cells show a high propensity to form >4N cells due to radiation alone, with NVP-AUY922 combined with radiation actually resulting in a decrease in the >4N population (Fig. 6D), with a concurrent increase in the G_1_ phase (Fig. S5B). NVP-AUY922 alone causes a minimal increase in subG_1_ populations for HN3 and no impact on the subG_1_ population above that already induced by radiation. HN5 cells exhibit a high >4N population under control conditions, one that remains unaltered by 4 Gy or NVP-AUY922. HN5 cells are highly sensitive to NVP-AUY922-induced increase in subG_1_ with radiation and NVP-AUY922 combinations resulting in similar subG_1_ values to radiation-free drug concentrations.

## Discussion

Use of HSP90 inhibition as an approach to radiosensitization was first investigated by Tofilon and colleagues. 17-AAG was shown to radiosensitize prostate cancer and glioma cell lines exhibiting the now familiar molecular signature [20], [33] of depletion of radioresistance markers and HSP90 client proteins AKT, ErbB2 and cRAF [8]. Since then, alternative HSP90 inhibitors have been developed including other geldanamycin derivates such as 17-DMAG, IPI-504 and fully synthetic inhibitors such as BIIB021, SNX-5422, STA-9090 and KW-2478. Thus far, radiosensitization effects by this group of compounds have been confined to geldanamycin family compounds and the purine analog BIIB021. Geldanamycin or its analogs have been shown to radiosensitize *in vitro* an array of cancer cell lines from many tissue origins [9], [11]–[14], [16], [18], proving the broad therapeutic applicability of HSP90 inhibitors as radiosensitizers.

Numerous studies have verified client protein depletion at radiosensitizing concentrations of geldanamycin [13], [14], [16], 17-AAG [11], [18], 17-DMAG [9], [12] and BIIB021 [10]. This is also true for NVP-AUY922, with depletion of radioresistant client proteins ErbB2, AKT and cRAF occurring at 5–10 nM for HeLa and HN3 cell lines (Fig. 3A), with depletion of total-ErbB2 and decreasing phospho-ErbB2 signalling identifying it as the HSP90 client protein most effected by NVP-AUY922.

The effective radiosensitization dose *in vitro* in the literature ranges from 50–200 nM for geldanamycin [13], [14], [16], 30–200 nM for 17-AAG [11], [16], [18], 10–50 nM for 17-DMAG [9], [12] and 125–200 nM for BIIB021 [10]. The effective radiosensitization doses of 1–5 nM shown in Figure 1 indicate NVP-AUY922 to be the most potent *in vitro* HSP90-targeted radiosensitizer yet reported.

Only three previous studies have described *in vivo* radiosensitization by HSP90 inhibitors. These have included studies of cervical, prostate and HNSCC human tumor xenograft models with varying dose and radiation schedules. 17-AAG has been used in single and multiple doses of 150 and 125 mg/kg, respectively, with single fractions of 12 Gy and fractionated radiation (5×2 Gy) [16]. 17-DMAG was administered in two 50 mg/kg doses with 5 Gy given 12 h after final dosing [12]. BIIB021 and 17-AAG were administered three times weekly with 1.5 Gy given four times per week for the 4–5 week experimental duration [10]. In all previously published experiments, drug alone groups caused significant tumor regression in their own right. The results shown in Figure 2 for 10 mg/kg NVP-AUY922 are indicative of a true radiosensitizer with similar tumor volumes for both drug and control groups at the doses used and a significant radiosensitization effect observed for drug plus radiation compared with radiation alone. Significantly increased median time to surrogate survival endpoint was observed for NVP-AUY922 combined with radiation over radiation alone and to our knowledge this is the first instance where survival data have been reported alongside tumor volume data for any HSP90-based radiosensitizer. Decrease in tumour volume has been shown previously at 10–20 mg/kg for both BIIB021 and 17-AAG [10]. However, treatment was initiated at tumor diameter of 2–3 mm, substantially lower than the tumor volume used in this study.

While this is the first publication of NVP-AUY922 as a radiosensitizer *in vivo*, it is also important to note that to our knowledge this is the first validation of *in vivo* radiosensitization by a HSP90 inhibitor with a high degree of selectivity for HSP90α and HSP90β over other HSP90 family isoforms. In comparable competitive binding fluorescence polarisation assays, IC_50_ values have been shown to be; HSP90β 21±6 nmol/L, GRP94 540±29 nmol/L, TRAP1 850±4 nmol/L for NVP-AUY922 [36]; HSP90 (alpha or beta form not stated) 119±23 nM and GRP94 124±53 nM for 17AAG [37] (TRAP1 not tested). BIIB021 binding assays for other HSP90 family isoforms have not been reported in the literature. Additionally, *in vitro* radiosensitization by 100 nM and 200 nM NVP-AUY922 has been reported previously and was said to be similar to that of 17-DMAG [38], [39]. This is in contrast to the substantially increased potency observed *in vitro* in the present study and with the substantially increased potency of NVP-AUY922 over geldanamycin analogs by other metrics of HSP90 inhibition [20], [28], [31], [40]. Increased potency over geldanamycin family analogues is a property of NVP-AUY922 which has been clearly shown for drug only effects but has been missed in previous radiosensitization studies. This difference can be attributed to NVP-AUY922 administration post-radiation [38], [39], while early studies showed the efficacy of HSP90 mediated radiosensitization was time-dependent on pre-treatment [12], [16] explaining the significant increase in radiosensitization potency observed in this study.

Pharmacokinetic studies of NVP-AUY922 *in vivo* in human melanoma and colorectal carcinoma xenografts models have shown that 50 mg/kg NVP-AUY922 administered on five consecutive days is capable of maintaining a dose of 3.8 to 7.7 µmol/L in tumor tissue over 24 hours [20]. *In vitro* uptake of 80 nM NVP-AUY922 for HCT116 cells equates to internal cell concentrations of 5 µmol/L, a concentration shown to be achievable *in vivo* [20]. If cellular uptake of NVP-AUY922 by HN3 cells is in a similar range to HCT116 cells, it can be assumed that the *in vivo* equivalent of 10–20 nM concentration shown to exhibit the molecular markers of radiosensitization (Figures 3, 4, 5, 6) is achievable at dosing of 10 mg/kg based on similar calculations. This is notwithstanding the potential radiosensitization-independent benefits of NVP-AUY922 previously shown, such as improvement in lung metastasis in terms of number, size and lung volume occupied in BRAF mutant WM266.4 melanoma xenografts, absence of lymph node metastasis in PTEN-null human prostate carcinoma (PC3LN3) model and decreased microvessel density in U87G PTEN-null glioblastoma xenografts [20].

*In vitro* experiments have shown that HSP90 inhibitors do not radiosensitize non-cancer cell lines [8], [14], [16], [17], [41]. In areas not subject to radiation, but still exposed to NVP-AUY922, a clear therapeutic window has been demonstrated between peripheral blood mononuclear cells and primary multiple myeloma cells. In addition, a return to active proliferation of bone marrow stromal cells upon drug withdrawal has been seen even after 72 h treatment with 200 nM NVP-AUY922 [30].

Single dose exposure to radiation alone was found to cause minimal upregulation of HSP72 *in vitro* in HeLa cells or the two HNSCC cell lines, HN3 or HN5, with any effect of radiation in combination with NVP-AUY922 masked by predominant HSP90 inhibition-induced HSP72 upregulation (Fig. 3C, D; Fig. S2). However, fractionated radiation with repeated dosing of NVP-AUY922 did show upregulation *in vivo*, an effect that was seen with neither radiation nor NVP-AUY922 alone. The occurrence of this disparity is unsurprising, considering the substantially greater degree of cellular stress that can be delivered *in vivo* due to the combination of multiple drug administrations and fractional radiation dosing over a number of consecutive days. This is, however, a factor that must be taken into consideration by virtue of the potential for HSP72-mediated anti-apoptotic effects to reduce radiosensitization through HSP90 inhibition.

Upregulation of HSP72 by heat shock has been shown to be protective from UVB [42], with depletion of HSP72 shown to enhance the sensitivity of cells to genotoxic stress such as radiation [43], [44]. Even so, subtherapeutic dosing of NVP-AUY922 was capable of mediating radiosensitization as measured by tumour growth delay and increased survival. Even if increased doses of NVP-AUY922 were administered as part of a regimen aiming for curative outcomes, it is likely that higher levels of HSP72 expression would still be counterbalanced by more potent HSP90 client protein depletion. Interestingly, evidence has shown that HSP72 mediates protective effects against UVC radiation through CHK1 [43]. Therefore, it is possible that potential anti-apoptotic effects due to HSP90 inhibitor-induced upregulation of HSP72 (as opposed to heat shock upregulation) may be significantly abrogated by depletion of HSP90 client proteins that are effectors of the anti-apoptotic process. Clearly, this is a important aspect of HSP90-mediated radiosensitization that is of great relevance to future clinical success and warrants further studies.

The increased pH2ax foci at 24 h in radiation plus NVP-AUY922 treated HeLa and HN3 cells (Fig. 4) demonstrates the ability of NVP-AUY922 to delay repair of radiation-induced DNA damage. This has previously been shown for 17-AAG in HCT116 p53 knockout, but not wild-type, cells [15] and has been suggested by alternative methods such as DNA fragment drop-out in gel electrophoresis [41]. The putative mechanism by which NVP-AUY922 achieves this delay is explained by findings that early HSP90 inhibitors deplete the critical homologous recombination proteins Rad51 [17], [41], [45], BRCA2 [17], [41] and impairs FANCD2 focus formation through upstream depletion of FANCA [5]. In all four cell lines tested, NVP-AUY922 at GI_50_ concentrations was found to reduce Rad51 foci 4 h post-radiation, with increased Rad51 foci 24 h post-radiation compared to radiation alone. These two readings taken together are indicative of a delay in Rad51 focal assembly post-radiation, leading to a decrease in the rate of successful homologous recombination, resulting in higher levels of residual Rad51 foci at 24 h. This was irrespective of active p53 (HCT116), EGF-R family overexpression (HN3, HN5) or activated kRAS and PI3K catalytic subunit mutations (HCT116). NVP-AUY922 has previously been reported not to deplete Rad51 [38]. However, our data conclusively show Rad51 focal formation is ubiquitously impaired and is putatively one of the underlying mechanisms for the broad radiosensitization observed for HSP90 inhibitors which occurs irrespective of a number of mutation states. This prolonged DNA repair can be hypothesised to increase the time period over which cells may trigger apoptosis as a result of DSB DNA damage and, when combined with CHK1 depletion (Fig. 6), it is also likely to increase the probability of an attempted cell cycle progression with an unacceptable/unsafe burden of DNA damage.

It has previously been shown that the G2/M checkpoint is less rigid than previously anticipated, with cells released once the number of DSBs drops below a threshold of approximately 20. A delay in homologous recombination by NVP-AUY922 on its own may simply lead to a prolonged G2/M arrest, as has been seen in cells defective in the DNA repair nuclease Artemis [46]. The ability to concurrently abrogate both HR and arrest, forcing mitosis with a DSB level significantly higher than the threshold level is likely to be the cause of the increased chromosomal breakage shown (Fig. 5A; Fig S4). It has previously been observed that dual deficiency in both checkpoint arrest and DNA repair results in an increased number of chromosomal breaks than defects in DNA repair alone [47]. The ability of 17-AAG to radiosensitize homologous recombination-deficient cells [17] indicates the depletion of both CHK1 and radioresistance client proteins, such as those analysed in Fig. 3A, is also crucial to the cumulative radiosensitization effect.

Our data indicate that NVP-AUY922-induced radiosensitization attributable to cell cycle effects may not be ubiquitous, particularly in respect of HNSCC. While cell line-specific G_1_ arrest has been reported [10], most studies have shown that cells treated with HSP90 inhibitors in the absence of radiation accumulate in G_2_/M [20], [38], [48]–[50]. Maki and colleagues used the metaphase inhibitor colcemid as a mechanistic approach to show geldanamycin-induced abrogation of CHK1-mediated G_2_ arrest, as measured by M phase accumulation due to colcemid [15]. Data presented in Figure 6 indicate a substantial, but cell line-specific, accumulation (HeLa and HN5) in M-phase can be induced by NVP-AUY922 which is maintained in the presence of radiation damage, or even enhanced in the case of HeLa cells. The second group (HN3 and HCT116) do not display this accumulation over and above normal control mitotic index levels under any conditions.

It is unknown at this point if this is due to cell line-specific dependence on HSP90 chaperoned client proteins involved in mitotic progression, or depletion of client proteins such as topoisomerase IIα [45]. Morphological indicators of nuclear abnormalities (Fig. 5) indicate nuclear volume increases in HeLa and HN5 due to drug alone more than in HN3 or HCT116. Micronuclei, previously linked with radiosensitization by geldanamycin in HCT116 p53 and p21 knockout models [15] as well as more traditional radiosensitizers such as aurora B kinase inhibition [51], do not indicate any increase due to drug alone. The opposite is in fact true with HN3 and HCT116 showing higher levels of micronuclei due to drug alone. All four cell lines, however, show a clear increase in micronuclei formed due to NVP-AUY922 in combination with radiation.

HSP90-mediated depletion of CHK1 [15], [45], [49], [51] is clearly ubiquitous, with abrogation of G_2_ arrest leading to entry into mitosis even after irradiation [12], [15]. Cell cycle analysis at 48 h post-irradiation for HeLa and HCT116 are in line with expected results. Radiosensitization in HeLa cells corresponds to an increase in both >4N and subG_1_ population (Fig. 6), while the p53-positive HCT116 shows resistance to cell cycle perturbation at 48 h. Results for HN3 and HN5 are more difficult to interpret. Little observable difference is seen for HN5 which is highly sensitive to sub-G_1_ accumulation due to NVP-AUY922 alone (Fig. 6; Fig. S5B). HN3 cells have a high baseline >4N population, with NVP-AUY922 actually reducing radiation-induced >4N cells, corresponding to an increase in the G_1_ cell population. How this NVP-AUY922-mediated intervention in radiation-induced >4N population formation may contribute, either positively or negatively, to survival is not known. Unlike the ubiquitous effects observed for client protein depletion, DSB repair and chromosomal fragmentation, cell cycle data for NVP-AUY922 in combination with radiation is significantly more variable across cell lines. Cell cycle data suggests the outcome of the ubiquitous HSP90 effects indicated may not result in a uniform radiosensitization linked end point, this represents an aspect of radiosensitization which warrants further study in HNSCC cell lines.

The present preclinical work shows that NVP-AUY922 is a promising candidate for translational clinical studies in conjunction with external beam radiotherapy in head and neck squamous cell carcinoma. The current body of literature strongly supports the potential of HSP90 inhibition to enhance the therapeutic window between normal tissue and that of malignant neoplasms. Of potential substantial benefit is the diversity of downstream effects mediated via a single targeted agent. In the sphere of chemoradiotherapy, the benefit of simultaneously disabling prosurvival pathways, checkpoint arrest and DNA damage repair is one of considerable promise in improving clinical outcomes. Although considered less of a concern in this multi-targeted approach, ErbB3 expression (which has been shown to be overexpressed in HNSCC) has been linked to resistance to 17-DMAG-induced radiosensitization [9]. In conjunction with cell cycle effects observed for the HNSCC cell lines HN3 and HN5 and cell line-dependent mitotic accumulation in HN5 and HeLa cells observed in this paper, this highlights the importance of continued investigation into the genetic and epigenetic factors related to HSP90-mediated radiosensitization. In conclusion, the data shown here identify NVP-AUY922 as the most potent HSP90-targeted radiosensitizer currently available both *in vitro* and *in vivo* and, as such, the most promising candidate studied to date for future clinical development.

## Supporting Information

Figure S1 **Tumor volumes corresponding to NVP-AUY922 for 10 mg/kg plus radiation in HN3 human HNSCC murine xenograft model.** (A) Clonogenic cell survival of HeLa, HCT116, HN3 and HN5 cell lines due to 24 h pre-treatment with 100 nM 17-AAG or NVP-AUY922 at the concentrations indicated followed by subsequent 2 Gy irradiation or mock-irradiation. Colony formation was determined at 10 to 14 days after irradiation and surviving fractions calculated relative to plating efficiencies for vehicle only non-irradiated cells. (B) HN3 cells were allowed to achieve a tumor volume of 5–8 mm after implantation in the right flank and evenly distributed into four treatment groups with matching average tumor volumes; vehicle only control (n = 8); NVP-AUY922, three doses of 10 mg/kg each (n = 9); 9 Gy ionising radiation fractionated in three doses of 3 Gy (n = 9); NVP-AUY922 and ionising radiation (n = 8). Actual tumor volumes used to calculate baseline tumor volumes in figure 1 are shown.
(PDF)

Figure S2 **HSP72 upregulation due to radiation in combination with NVP-AUY922.** (A) Triplicate western blot data used for densitometric quantitiation as shown in Figure 3B. (B) HN5 cells were pre-treated with NVP-AUY922 for 24 h at the concentrations indicated. Cells were irradiated with 4 Gy or 10 Gy and 4 h later whole cell lysates harvested and probed for HSP72 by western blot. (C) HeLa cells were irradiated with 4 Gy of radiation delivered at the 0 hour time point. At the subsequent time points indicated whole cell lysates were harvested and probed for HSP72. γ-tubulin and GAPDH were probed as loading controls as indicated.
(PDF)

Figure S3 **NVP-AUY922 delays Rad51 foci formation and resolution of phospho-H2ax foci.** HN5 and HCT116 cells were plated in glass bottom dishes and after attachment exposed to NVP-AUY922 or DMSO control. 24 h post drug-treatment cells were mock irradiated or irradiated with 4 Gy, 4 h and 24 h post-irradiation cells were fixed and stained for dsDNA breaks using anti-phospho-H2ax and anti-Rad51 with TO-PRO-3 as nuclear counter stain. (A) The average phospho-H2ax foci per-cell in HN5 and HCT116 cell lines at 4 h and 24 h post irradiation was quantified, with data shown for quantification of 150 cells in a single experiment. (B) Rad51 foci were quantified in HN5 and HCT116, with nuclei containing greater than 3 foci scored as positive. Foci formation in HN5 shown for one independent experiment and two for HCT116. (C) Quantification of average Rad51 foci per nuclei shown for HeLa, HN3, HN5 and HCT116 (calculated from the same dataset used in B above and Fig. 4C). HeLa and HN3 data shown as average of two independent experiments, HN5 and HCT116 data shown from one experiment.
(PDF)

Figure S4 **NVP-AUY922 in combination with radiation increases occurrence of pan-phospho-H2ax stained micronuclei in HN5 and HCT116 cell lines.** HN5 and HCT116 cells were treated with vehicle only or NVP-AUY922 as indicated for 24 h before mock irradiation or irradiation with 4 Gy. 24 h post-irradiation cells were fixed and stained for phospho-H2ax with TOPRO-3 as nuclear counterstain. Nuclei with associated pan-phospho-H2ax positive micronuclei were quantified for a minimum of 150 cells. Data shown as single experiment.
(PDF)

Figure S5 **NVP-AUY922 induces both increased entry and delayed exit from mitosis due to ionising radiation.** Cells were exposed to the NVP-AUY922 concentrations indicated for 16 h before mock irradiation or irradiation with 4 Gy. Cells were fixed at 9 h and 48 h post-irradiation before (A) staining for the mitotic marker phospho-histone H3 and (B) DNA content with propidium iodide and quantification of G_1_, S and G_2_ populations by FACS analysis. Data represents ± SEM of three independent experiments each recording at least 10,000 events.
(PDF)
