# Supplementary figures and images for: The HSP90 Inhibitor NVP-AUY922 Radiosensitizes by Abrogation of Homologous Recombination Resulting in Mitotic Entry with Unresolved DNA Damage
Source: PLoS One. 2012 Apr 16;7(4):e35436. doi: 10.1371/journal.pone.0035436 (PMC3327673; doi:10.1371/journal.pone.0035436)

**A**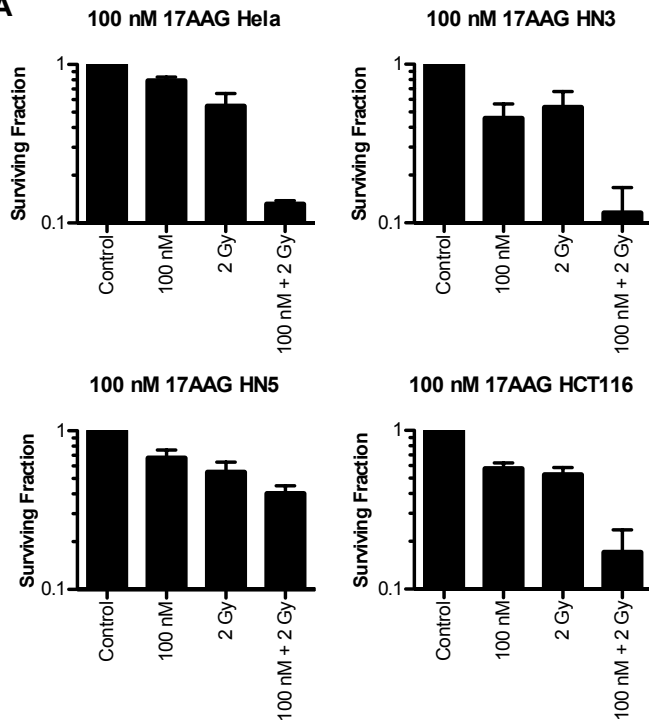**B**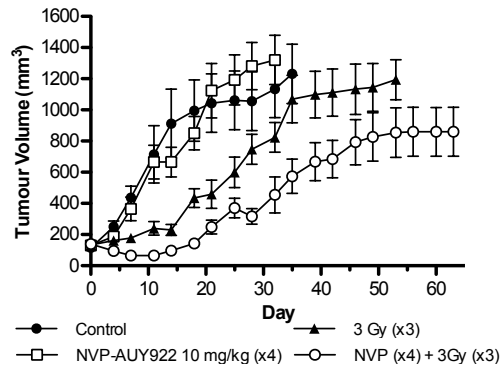

Supplement: Figure S1 — Tumor volumes corresponding to NVP-AUY922 for 10 mg/kg plus radiation in HN3 human HNSCC murine xenograft model. (A) Clonogenic cell survival of HeLa, HCT116, HN3 and HN5 cell lines due to 24 h pre-treatment with 100 nM 17-AAG or NVP-AUY922 at the concentrations indicated followed by subsequent 2 Gy irradiation or mock-irradiation. Colony formation was determined at 10 to 14 days after irradiation and surviving fractions calculated relative to plating efficiencies for vehicle only non-irradiated cells. (B) HN3 cells were allowed to achieve a tumor volume of 5–8 mm after implantation in the right flank and evenly distributed into four treatment groups with matching average tumor volumes; vehicle only control (n = 8); NVP-AUY922, three doses of 10 mg/kg each (n = 9); 9 Gy ionising radiation fractionated in three doses of 3 Gy (n = 9); NVP-AUY922 and ionising radiation (n = 8). Actual tumor volumes used to calculate baseline tumor volumes in figure 1 are shown. (PDF) [file pone.0035436.s001.pdf]

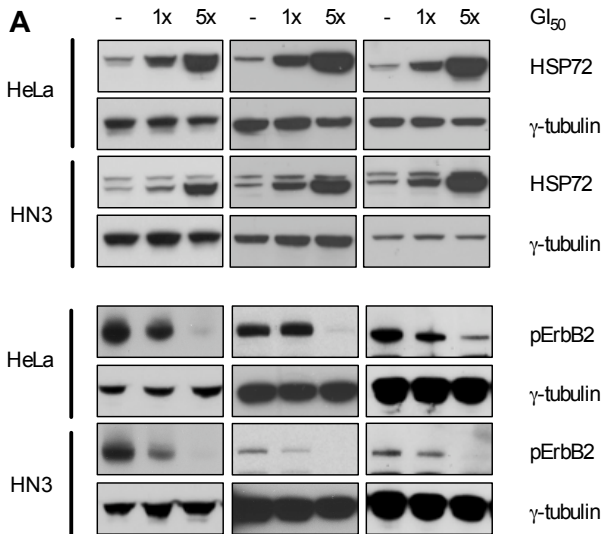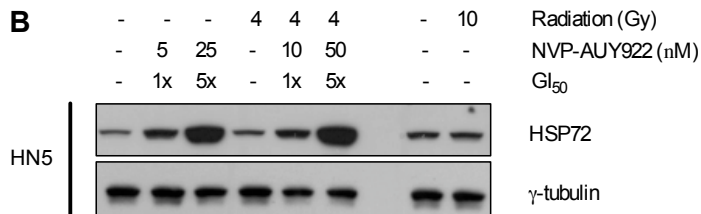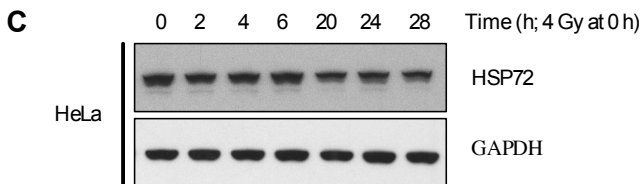

Supplement: Figure S2 — HSP72 upregulation due to radiation in combination with NVP-AUY922. (A) Triplicate western blot data used for densitometric quantitiation as shown in Figure 3B. (B) HN5 cells were pre-treated with NVP-AUY922 for 24 h at the concentrations indicated. Cells were irradiated with 4 Gy or 10 Gy and 4 h later whole cell lysates harvested and probed for HSP72 by western blot. (C) HeLa cells were irradiated with 4 Gy of radiation delivered at the 0 hour time point. At the subsequent time points indicated whole cell lysates were harvested and probed for HSP72. γ-tubulin and GAPDH were probed as loading controls as indicated. (PDF) [file pone.0035436.s002.pdf]

**A**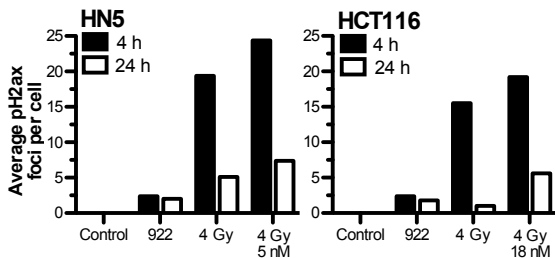**B**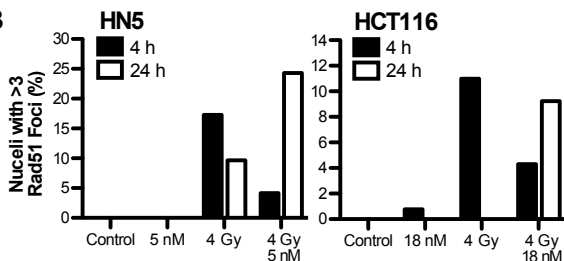**C**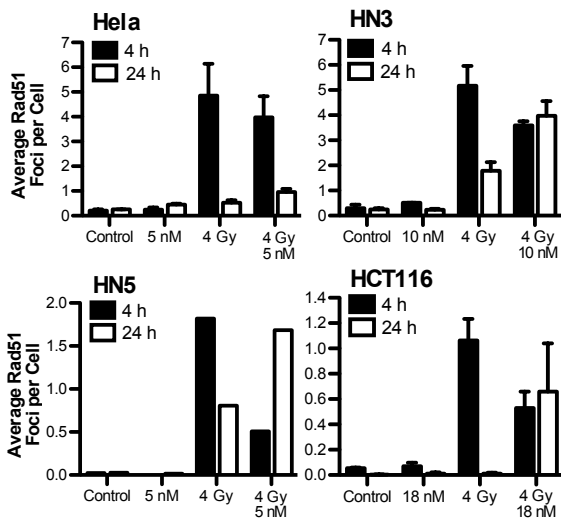

Supplement: Figure S3 — NVP-AUY922 delays Rad51 foci formation and resolution of phospho-H2ax foci. HN5 and HCT116 cells were plated in glass bottom dishes and after attachment exposed to NVP-AUY922 or DMSO control. 24 h post drug-treatment cells were mock irradiated or irradiated with 4 Gy, 4 h and 24 h post-irradiation cells were fixed and stained for dsDNA breaks using anti-phospho-H2ax and anti-Rad51 with TO-PRO-3 as nuclear counter stain. (A) The average phospho-H2ax foci per-cell in HN5 and HCT116 cell lines at 4 h and 24 h post irradiation was quantified, with data shown for quantification of 150 cells in a single experiment. (B) Rad51 foci were quantified in HN5 and HCT116, with nuclei containing greater than 3 foci scored as positive. Foci formation in HN5 shown for one independent experiment and two for HCT116. (C) Quantification of average Rad51 foci per nuclei shown for HeLa, HN3, HN5 and HCT116 (calculated from the same dataset used in B above and Fig. 4C). HeLa and HN3 data shown as average of two independent experiments, HN5 and HCT116 data shown from one experiment. (PDF) [file pone.0035436.s003.pdf]

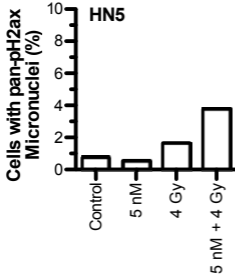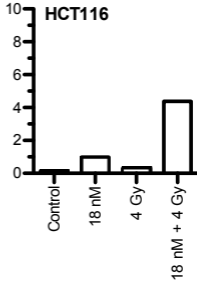

Supplement: Figure S4 — NVP-AUY922 in combination with radiation increases occurrence of pan-phospho-H2ax stained micronuclei in HN5 and HCT116 cell lines. HN5 and HCT116 cells were treated with vehicle only or NVP-AUY922 as indicated for 24 h before mock irradiation or irradiation with 4 Gy. 24 h post-irradiation cells were fixed and stained for phospho-H2ax with TOPRO-3 as nuclear counterstain. Nuclei with associated pan-phospho-H2ax positive micronuclei were quantified for a minimum of 150 cells. Data shown as single experiment. (PDF) [file pone.0035436.s004.pdf]

A

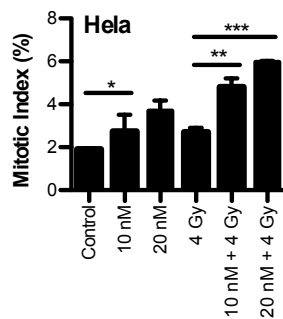

B

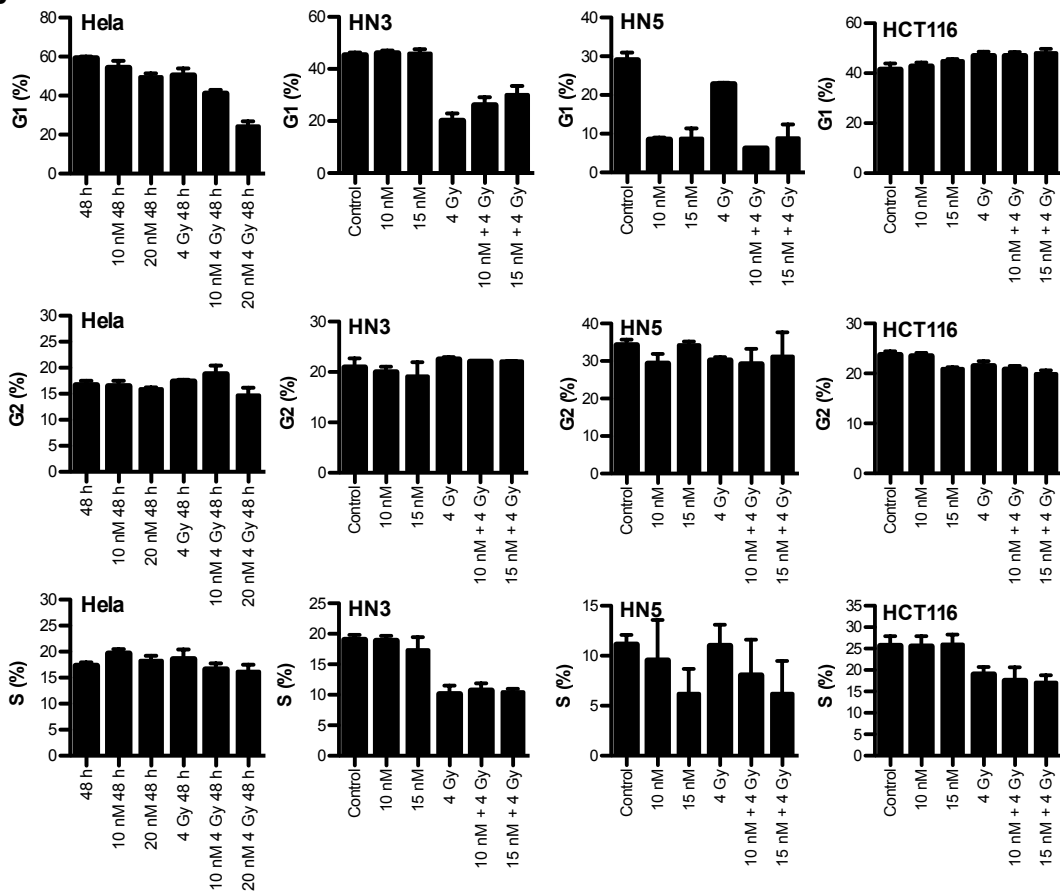

Supplement: Figure S5 — NVP-AUY922 induces both increased entry and delayed exit from mitosis due to ionising radiation. Cells were exposed to the NVP-AUY922 concentrations indicated for 16 h before mock irradiation or irradiation with 4 Gy. Cells were fixed at 9 h and 48 h post-irradiation before (A) staining for the mitotic marker phospho-histone H3 and (B) DNA content with propidium iodide and quantification of G1, S and G2 populations by FACS analysis. Data represents ± SEM of three independent experiments each recording at least 10,000 events. (PDF) [file pone.0035436.s005.pdf]
